# Management and outcome of topical beta-blockerinduced atrioventricular block

**DOI:** 10.5830/CVJA-2015-030

**Published:** 2015

**Authors:** Kazım Serhan Özcan, Barış Güngör, Ahmet İlker Tekkeşin, Servet Altay, Ahmet Ekmekçi, Ercan Toprak, Ersin Yıldırım, Nazmi Çalık, Ahmet Taha Alper, Kadir Gürkan, İzzet Erdinler, Damirbek Osmonov

**Affiliations:** Department of Cardiology, Derince Training and Research Hospital, Kocaeli, Turkey; Department of Cardiology, Siyami Ersek Cardiovascular and Thoracic Surgery Centre, Istanbul, Turkey; Department of Cardiology, Siyami Ersek Cardiovascular and Thoracic Surgery Centre, Istanbul, Turkey; Department of Cardiology, Siyami Ersek Cardiovascular and Thoracic Surgery Centre, Istanbul, Turkey; Department of Cardiology, Siyami Ersek Cardiovascular and Thoracic Surgery Centre, Istanbul, Turkey; Department of Cardiology, Siyami Ersek Cardiovascular and Thoracic Surgery Centre, Istanbul, Turkey; Department of Cardiology, Siyami Ersek Cardiovascular and Thoracic Surgery Centre, Istanbul, Turkey; Department of Cardiology, Siyami Ersek Cardiovascular and Thoracic Surgery Centre, Istanbul, Turkey; Department of Cardiology, Siyami Ersek Cardiovascular and Thoracic Surgery Centre, Istanbul, Turkey; Department of Cardiology, Siyami Ersek Cardiovascular and Thoracic Surgery Centre, Istanbul, Turkey; Department of Cardiology, Siyami Ersek Cardiovascular and Thoracic Surgery Centre, Istanbul, Turkey; Department of Cardiology, Almaty Sema Hospital, Almaty, Kazakhstan

**Keywords:** beta-blockers, glaucoma, drug-induced block, pacemaker implantation

## Abstract

**Background:**

Topical beta-blockers have a well-established role in the treatment of glaucoma. We aimed to investigate the outcome of patients who developed symptomatic atrioventricular (AV) block induced by topical beta-blockers.

**Methods:**

All patients admitted or discharged from our institution, the Siyami Ersek Training and Research Hospital, between January 2009 and January 2013 with a diagnosis of AV block were included in the study. Subjects using ophthalmic beta-blockers were recruited and followed for permanent pacemaker requirement during hospitalisation and for three months after discontinuation of the drug. A permanent pacemaker was implanted in patients in whom AV block persisted beyond 72 hours or recurred during the follow-up period.

**Results:**

A total of 1 122 patients were hospitalised with a diagnosis of AV block and a permanent pacemaker was implanted in 946 cases (84.3%) during the study period. Thirteen patients using ophthalmic beta-blockers for the treatment of glaucoma and no other rate-limiting drugs were included in the study. On electrocardiography, eight patients had complete AV block and five had high-degree AV block. The ophthalmic beta-blockers used were timolol in seven patients (55%), betaxolol in four (30%), and cartelol in two cases (15%). The mean duration of ophthalmic beta-blocker treatment was 30.1 ± 15.9 months. After drug discontinuation, in 10 patients the block persisted and a permanent pacemaker was implanted. During follow up, one more patient required pacemaker implantation. Therefore in total, pacemakers were implanted in 11 out of 13 patients (84.6%). The pacemaker implantation rate did not differ according to the type of topical beta-blocker used (*p* = 0.37). The presence of infra-nodal block on electrocardiography was associated with higher rates of pacemaker implantation.

**Conclusion:**

Our results indicate that topical beta-blockers for the treatment of glaucoma may cause severe conduction abnormalities and when AV block occurs, pacemaker implantation is required in a high percentage of the patients.

## Background

Topical beta-blockers have a well-established role in the treatment of glaucoma and are frequently used as first-line therapy for the reduction of associated ocular hypertension.^1^,^2^ While systemic concentration after administration of topical beta-blockers is low in comparison to that achieved with oral beta-blockers, cardiovascular, respiratory, central nervous system and metabolic side effects may still occur.^3^ Topical beta-blockers have been shown to decrease heart rate and blood pressure in comparison to placebo.^4^ Cardiovascular effects may be augmented with systemic combination therapy with other heart rate-blocking agents, such as beta-blockers and calcium channel blockers.^5^

In the literature, there are several case reports indicating the possible relationship between topical beta-blockers and the development of severe bradyarrhythmias, such as third-degree atrioventricular (AV) block and sick sinus syndrome.^6^-^9^ However, little is known about the incidence and prognosis of severe bradyarrhythmias induced by topical beta-blockers. In this trial, we aimed to investigate the outcome of patients who were hospitalised with a diagnosis of symptomatic AV block while receiving topical beta-blockers.

## Methods

All patients who were hospitalised in our institution, the Siyami Ersek Training and Research Hospital, between January 2009 and January 2013 with a diagnosis of AV block or symptomatic bradyarrhythmia were reviewed. The site of AV block was diagnosed by surface electrocardiography, as previously described^10^-^13^ (Table 1). Patients who had symptomatic (fatigue, faintness, dyspnoea and syncope) type II second- or third-degree AV block, 2:1 AV block, atrial fibrillation with bradycardia (average heart rate ≤ 40 beats/min on 24-hour Holter monitoring) were included in this study. Patients with vasovagal syncope, concomitant myocardial infarction, electrolyte abnormalities or digitalis toxicity were excluded.

**Table 1:** Classification of second- and third-degree AV block, and atrial fibrillation with bradyarrhythmia, based on electrocardiographic characteristics

	*AV nodal block*	*Infra-nodal AV block*	*Undetermined level of AV block*
Second-degree AV block	PR increment preceding a blocked P (Wenckebach) and narrow QRS	Constant PR interval preceding blocked P	PR increment (Wenckebach) preceding a blocked P and wide QRS
2:1 AV block	Conducted impulse has long PR and narrow QRS; PR varies inversely with RP	Conducted impulse has normal PR and wide QRS; PR is constant despite varying RP	Conducted impulse has long PR and wide QRS or short PR and narrow QRS
Third-degree AV block	Escape rhythm has narrow QRS and rate ≥ 40 beats/min	Escape rhythm has wide QRS and rate < 40 beats/min	Escape rhythm has wide QRS and rate ≥ 40 beats/min
Atrial fibrillation and bradyarrhythmia	f waves with irregular narrow QRS	f waves with regular wide QRS	f waves with irregular wide QRS

AV = atrioventricular

Subjects using ophthalmic beta-blockers were selected and followed for permanent pacemaker requirement during the hospitalisation period and for three months after discontinuation of the drug. Topical beta-blockers were discontinued after the initial refererral to the hospital. According to the response of the AV conduction after drug withdrawal, the type of adverse drug reaction was identified from the definition of ‘adverse drug reactions’ reported by Edwards and Aronson.^14^

A permanent pacemaker was implanted in patients in whom AV block persisted beyond 72 hours or recurred during the follow-up period. All of the patients were referred to their primary physicians for treatment of glaucoma after discharge.

## Statistical analysis

All data were presented as mean ± SD for parametric variables and as percentages for categorical variables, unless stated otherwise. Categorical variables were analysed with the Pearson’s χ^2^ test and Fisher’s exact test. All statistical studies were carried out using Statistical Package for Social Sciences software (SPSS 16.0 for Windows, SPSS Inc, Chicago, Illinois) and a *p*-value < 0.05 was considered statistically significant.

## Results

A total of 1 122 patients were hospitalised with a diagnosis of AV block and a permanent pacemaker was implanted in 946 cases (84.3%). Thirteen of the 1 122 patients (1.1%) were using ophthalmic beta-blockers for the treatment of glaucoma. The demographic and clinical characteristics of these patients are summarised in Table 2. None of these 13 patients were using rate-limiting agents (oral beta-blockers, non-dihydropyridine calcium channel blockers, digoxin and anti-arrhythmic drugs).

**Table 2:** Characteristics of patients with ophtalmic beta-blocker-induced conduction defects

*Patient’s age/gender*	*Drug type*	*Therapy duration (months)*	*Conduction defect*	*Temporary/permanent pacemeker*
81/M	Betaxolol	24	3rd-degree AV block	Yes/Yes
58/F	Timolol	40	Sinus pause	No/Yes
78/F	Timolol	52	3rd-degree AV block	Yes/No*
82/M	Timolol	14	High-degree AV block	No/Yes
85/M	Timolol	15	3rd-degree AV block	No/Yes
62/F	Timolol	61	Sinus pause	No/No*
56/F	Timolol	44	3rd-degree AV block	No/Yes
62/M	Cartelol	32	High-degree AV block	No/Yes**
73/M	Betaxolol	16	3rd-degree AV block	No/Yes
83/F	Betaxolol	18	High-degree AV block	No/Yes
72/M	Cartelol	11	3rd-degree AV block	Yes/Yes
65/F	Betaxolol	37	High-degree AV block	No/Yes
76/M	Timolol	27	3rd-degree AV block	No/Yes

*Rhythm was improved and conduction disturbance never recurred after drug withdrawal; **Rhythm was improved but recurred one month after drug discontinuation. AV: atrioventricular.

The mean age was 71.7 ± 10.1 years and six patients in this group were male (46%). Nine patients had hypertension (69%) and four (30%) had coronary artery disease. The mean left ventricular ejection fraction was 58.4 ± 11.1% in the study population. The major symptoms were syncope in five subjects, dizziness in four, and bradyarrhythmia-related dyspnoea in four. The ophthalmic beta-blocker used was timolol in seven cases (55%), betaxolol in four (30%), and cartelol in two (15%). The mean duration of ophthalmic beta-blocker treatment was 30.1 ± 15.9 months.

On ECG, eight patients had complete AV block and five had high-degree AV block. The level of conduction block, according to ECG criteria, was as follows: four patients on betaxolol (100%) had infra-nodal block; two on cartelol (100%) had undetermined rhythm, four on timolol had infra-nodal block (57%), and three (43%) on timolol had AV node block.

After drug discontinuation, in 10 patients the block persisted and a permanent pacemaker was implanted. Three patients (two on timolol and one on cartelol therapy), in whom the AV block had resolved, were discharged without pacemaker implantation. In one patient on previous cartelol treatment, the AV block reccurred one month after hospital discharge and a permanent pacemaker was implanted. Therefore, in total, 11 of 13 patients required permanent pacemaker implantation (84.6%). The implantation rate did not differ according to the type of topical beta-blocker used (*p* = 0.37).

The distribution of ophthalmic beta-blockers in those who required permanent pacemaker implantation were timolol in five cases, betaxolol in four, and cartelol in two cases. The level of conduction block in patients who required permanent pacemaker implantation were infra-nodal block in eight cases, AV node block in one case and undetermined level of block in two cases. Only two patients on timolol therapy, whose ECGs were compatible with AV node block, did not require pacemaker implantation. On the other hand, all of the subjects with infranodal block on ECG required pacemaker implantation.

## Discussion

Drug-induced AV block is not a well-known clinical entity and there are controversial reports in the literature.^15^,^16^ Moreover, little is known about the natural history and prognosis of patients with drug-induced AV block on treatment with topical beta-blockers. The main finding of our study was that most of the patients with topical beta-blocker-induced AV block needed pacemaker implantation during follow up.

Topical beta-blockers decrease intra-ocular pressure by reducing the inflow of aqueous humour, which is controlled by the adrenergic system in the ciliary epithelium.^17^ However, only two to 10% of the drugs may penetrate to the inner parts of the eye and the periocular tissues.^18^ The remainder of the drugs (about 80% for timolol) enter the systemic circulation through the conjunctival vessels and via the nasolacrimal duct through the nasal mucosa, and reach peak plasma concentrations within five to 30 minutes.^19^,^20^

Quaranta *et al.* showed that timolol significantly reduced systolic and diastolic blood pressures.^21^, ^22^ Beta-blockers decrease sinus node automaticity, prolong sino-atrial, intra-atrial and atrioventricular conduction times, and increase atrioventricular node refractoriness.^23^ Orzalesi *et al.* reported that the level of cardiovascular risk was significantly higher in glaucoma patients,^24^ although in potentially predisposed patients, topical beta-blockers may cause adverse cardiovascular events secondary to systemic effects.

In a randomised trial conducted in glaucoma patients using placebo, topical beta-blockers or dual therapy of topical and oral beta-blockers, the pulse rate was significantly lower in the topical beta-blocker group compared to the controls (70.3 vs 76 beats/min). Heart rate was lowest in the dual-therapy group, which was reported as 58 beats/min.^5^ In our study, even though none of the patients received dual beta-blocker therapy, severe conduction disturbances were observed in patients using topical betablockers. In most of the patients, even after discontinuation of the drug, the conduction disturbances persisted and permanent pacemaker implantation was required.

In a previous study by our group, permanent pacemaker implantation rate was 48% in patients taking rate-limiting drugs except topical beta-blockers.^16^ However, in this cohort, the rate of permanent pacemaker implantation was significantly higher (84.6%, *p* = 0.01).

The mean age of patients in earlier and more recent articles was similar (72.01 vs 71.7 years, respectively).^15^ However, the level of AV block was significantly different in two articles: 28 of 108 patients (25.9%) taking rate-limiting drugs except topical betablockers had infra-nodal AV block, while eight of 13 patients (61.5%) on topical beta-blockers had infra-nodal AV block.^15^

In previous trials conducted on patients with drug-induced AV block, it was shown that infra-nodal block was associated with a higher rate of pacemaker implantation.^15^,^16^ Topical betablockers may not disturb electrical conduction as strongly as oral rate-limiting drugs due to their lower dose and the route of administration. However, topical beta-blockers may induce AV block in susceptible patients, and there are case reports of permanent pacemaker implantation in patients receiving them.^6^-^8^

Edwards and Aronson classified adverse drug reactions into six types: dose related (augmented), non-dose related (bizarre), dose related and time related (chronic), time related (delayed), withdrawal (end of use), and failure of therapy (failure).^14^ In our cohort, AV conduction fully recovered after drug withdrawal in two patients who were on fixed-dose timolol for more than four years. In 11 of 13 patients, AV conduction did not recover after drug withdrawal, which means the dose and length of time it was administered had no effect. According to the above drug-reaction classification, we concluded that 11 of 13 of our patients had non-dose-related (bizarre) drug reactions, and two of 13 patients had time-related (delayed) drug reactions. We suggest, however, that if AV block occurs during treatment with ophthalmic beta-blockers, it may be an indicator of an underlying severely damaged electrical pathway.

Lopez *et al.* evaluated the prognosis of 12 patients with ophthalmic beta-blocker-induced AV block.^8^ In their series, seven of the 12 patients recovered during follow up and five needed pacemaker implantation. They concluded that every patient with AV block must be questioned about concomitant use of eye drops. The pacemaker implantation rate in our series was higher than in their series, but the percentage of patients using topical beta-blockers was higher in their series (12 of 243 patients with AV block vs 13 of 1 122 patients with AV block in our series). In their study the age, gender, presence of a bundle branch block, escape rhythm on ECG, or dosage of the drugs did not predict the risk for permanent pacemaker implantation.^8^

## Limitations

We evaluated elderly symptomatic patients who required hospitalisation in a tertiary centre. We did not include out-patient clinic patients, asymptomatic cases, those with mild symptoms or patients with transient forms of conduction abnormalities in our study. This may explain the high rate of pacemaker implantation in our cohort, which included patients with more severe forms of conduction abnormalities secondary to topical beta-blocker therapy. Irrespective of the accuracy of the electrocardiographic characteristics in defining the level of the AV block, His-bundle recording was not performed during the course of this study and we could also not determine a causal relationship.

## Conclusion

This study and previous reports show that patients using topical beta-blockers may suffer severe bradyarrhythmias secondary to AV block, and a significant percentage of these cases required pacemaker implantation. Patients with underlying pathology of the sinus or AV node, or the conduction pathways may develop AV block while on treatment with ophthalmic beta-blockers and these drugs may reveal concealed AV block. It may therefore be beneficial to search for pre-existing conduction abnormalities in patients with glaucoma before initiation of this type of medication.
